# “Homœopathy” a Trade Mark

**Published:** 1886-01

**Authors:** Henry K. Leake


### CORRESPONDENCE.

### "HOMŒOPATHY"—A TRADE MARK.

TO THE EDITOR OF DANIEL'S MEDICAL JOURNAL:

*Dear Sir:—*

THE strictures on the fallacious theories and defective morality of the so-called homœopathic profession, which in your last issue are so forcibly indulged will be considered by all reflecting minds as being well deserved.

You may consistently claim, as you do by intimation, that the regular profession of medicine being a catholic, humane and enlightened body, which in its very nature it can only be, has never afforded to occupy a false position in morals before the public. Whatever may be inveighed against our short-comings in science and practice, which, notwithstanding our progress, we are willing to acknowledge yet remain in great number to annoy and humiliate us, the medical profession can never resort to subterfuge, or to ad-captandum and meretricious performances with the view of securing public patronage.

Now it is in these latter qualities wherein consists the "very head and front of the offending," expressed in the *titular device* of the homœopath, the *corpus delicti* of the crime against society of which he is guilty, and compared with which his other faults are mere peccadilloes.

To privately confess, when pressed, that experience has certainly demonstrated that "there can be no exclusive method of cure," which admission is a positive desertion of the Hahnemannian creed, and then surreptitiously practice on the principle, but advertise the contrary under a *trade mark* is, as you insist, the very climax of charlatanry and immorality.

The therapeutical non-sensicalities which were promulgated by Hahnemann, and which were elaborated from a principle distinctly plagiarized by him, could well be tolerated now, as they were before the advent of the would-be reformer, by a liberal-minded profession. There are many honest differences of opinion amongst us, and on account of them, we are often adjudged foolish in the extreme by our fellows. The platform of the medical profession, however, is broad enough for the most heterodox practitioners, as well as for those who imitate their predecessors or contemporaries. No man's reason or independence is here subjugated. Each one ascertains the facts for himself, as best he can, and then, as Mr. Locke says, "bottoms" on them. From so sure a basis he may generalize at pleasure according to the powers with which nature and education have endowed him. But his results, be they ever so startling or beneficient, he has no right to patent, as it were, by a *trade device* which it is evident degrades not only the spirit, but the very purpose of scientific investigation.

It is both instructive and gratifying to note that prominent homœopathic practitioners of London, who, having been brought to see the injustice of the distinction sought to be conveyed by Hahnemann and his deciples in the terms "Homœopath" and "Allopath," now drop their trade cognomen. It is no less significant, also, that the influential homœopathic journal of New York has abolished the name from its title page. Let us hope that this reform will continue to grow until the entire homœopathic body will be expurgated of at least all reproach to their moral code, which reflects upon their intelligence. Let those who indulge it—and they are many—renounce the unseemly attitude of the sycophant and professional mendicant and forego the pulings of the self-appointed martyr; and let them bring to their aid the honorable impulses of the conscientious worker in the medical vineyard, where the harvest will be garnered, not by one man, nor *so-called school of men*, but by *all* who "learn to labor and to wait."

Respectfully,

HENRY K. LEAKE, M.D.

